# Energy-Based Approach for Fatigue Life Prediction of Additively Manufactured ABS/GNP Composites

**DOI:** 10.3390/polym17152032

**Published:** 2025-07-25

**Authors:** Soran Hassanifard, Kamran Behdinan

**Affiliations:** Advanced Research Laboratory for Multifunctional Lightweight Structures (ARL-MLS), Department of Mechanical and Industrial Engineering, University of Toronto, Toronto, ON M5S 3G8, Canada

**Keywords:** additive manufacturing, fatigue life, energy-based approach, notch factor, ABS/GNPs composites

## Abstract

This study examines the effectiveness of energy-based models for fatigue life prediction of additively manufactured acrylonitrile butadiene styrene (ABS)/graphene nanoplatelet (GNP) composites. The effects of varying GNP weight percentages and filament raster orientations on the fatigue life of the samples were investigated theoretically. The required stress and strain values for use in energy-based models were obtained by solving two sets of Neuber and Ramberg–Osgood equations, utilizing the available values of notch strength reduction factors at each load level and the average Young modulus for each composite material. Results revealed that none of the studied energy-based models could accurately predict the fatigue life of the samples across the entire high- and low-cycle fatigue regimes, with strong dependence on the stress ratio (R). Instead, a novel fatigue life prediction model was developed by combining two existing energy-based models, incorporating stress ratio dependence for cases with negative mean stress. This model was tested for R values roughly between −0.22 and 0. Results showed that, for all samples at each raster orientation, most of the predicted fatigue lives fell within the upper and lower bounds, with a factor of ±2 across the entire range of load levels. These findings highlight the reliability of the proposed model for a wide range of R values when mean stress is negative.

## 1. Introduction

The advancement of Additive Manufacturing (AM) technologies, commonly known as 3D-printing, has enabled the fabrication of complex geometry components across various industries. Fused Deposition Modelling (FDM), a common AM technique, is extensively utilized to produce such components, offering versatility in materials and size scales [1]. In FDM, a filament is heated above its melting point, extruded through a nozzle, and deposited onto a preheated bed to form the desired geometry [2,3]. The quality and durability of FDM-produced parts depend on various parameters, including temperature, material viscosity, printing speed, layer height, and fill density [4,5]. Additionally, factors such as raster orientation, variations in the mechanical properties of the filaments, and inherent porosity significantly influence the mechanical properties of these components [6,7]. Raster orientation refers to the direction of filament deposition in FDM printing, typically defined by the angle relative to the loading direction. Careful consideration of these processing parameters is essential to enhance the reliability and performance of FDM-fabricated parts.

There is a growing need to investigate the durability and reliability of AM-produced products, particularly their fatigue resistance. A comprehensive literature review reveals that most studies on the fatigue behavior of AM parts focus on experimental statistical analysis, while fatigue life prediction methods remain relatively underexplored [8,9]. Among the existing studies in the literature, Mirzaei et al. [10] proposed a strain-based finite fracture mechanics model to predict the fatigue life of additively manufactured notched components under uniaxial loading. The model combines a non-local strain criterion with an energy balance condition and uses inputs obtained from strain-life and stress intensity factor-life data. Validation against experimental data for various materials, raster orientations, and notch geometries demonstrated that this model outperforms traditional approaches, such as classical finite fracture mechanics and the theory of critical distances [10]. While experimental studies offer valuable information into the factors influencing fatigue performance, they often require substantial data, making them costly and time-consuming. Consequently, researchers have increasingly turned to fatigue damage models to predict the fatigue life of components efficiently.

So far, energy-based fatigue life prediction models have been widely used to estimate the fatigue life of different metal and alloys [11,12,13,14,15]. In this approach, the total dissipated strain energy density—comprising elastic and plastic strain energy densities—is correlated with fatigue life. However, existing models differ in their definitions of elastic strain energy density (ESD), leading to ongoing debate on the best approach to account for mean stress effects and accurately predict fatigue life. To address this, various definitions of the positive ESD term and different computational techniques have been proposed [16,17]. Roostaei et al. [18] compared several models, highlighting the potential advantages of energy-based methodologies. Tao and Xia [19] used strain energy density under varying mean stresses and stress amplitudes to evaluate the fatigue life of an epoxy polymer, demonstrating that this approach is equally effective for predicting the fatigue life of plastic components.

Research on fatigue life prediction models for materials fabricated through AM techniques is limited, particularly for 3D-printed thermoplastics [20,21]. This study aims to explore the application of energy-based models for predicting the fatigue life of FDM-fabricated acrylonitrile butadiene styrene (ABS) composites reinforced with graphene nanoplatelets (GNPs), focusing on the effects of varying GNP content and raster orientation. To achieve this, key energy-based fatigue parameters—including the fatigue strength coefficient and exponent as well as the fatigue toughness coefficient and exponent—were derived from existing fatigue test data for the filament materials. These parameters were incorporated into several established energy-based models for fatigue life prediction.

To address the inherent porosity of FDM-produced parts, the anisotropic 3D-printed samples were modeled as homogeneous isotropic specimens with an imaginary notch, characterized by a notch strength reduction factor to account for stress concentration effects. The influence of three distinct raster angles (0°, 45°, and 90°) was investigated to assess the impact of printing orientation on fatigue life. A key novelty of this study lies in the development of a new energy-based fatigue life prediction model that is dependent on the stress ratio ($R=\sigma_{min}/\sigma_{max}$) and combines two existing models to more accurately handle cases with negative mean stress. The results obtained from the studied energy-based models showed that none accurately predicted fatigue life across the full range of R values. The proposed R-dependent hybrid model demonstrated superior predictive capability, showing good agreement with experimental data and addressing a major limitation in current fatigue prediction approaches for 3D-printed nanocomposites. In general, although the model was developed using ABS/GNP composites, it is based on general principles of energy-based fatigue modeling and stress/strain behavior near stress concentrators. Therefore, it can be extended to other FDM materials with similar microstructural features, particularly inter-filament voids and raster-induced anisotropy, such as PLA and Nylon.

## 2. Theoretical Background

Over the past few decades, several approaches have been developed for fatigue life predictions, which are mainly based on stress, strain, or energy [22]. During cyclic loading, micro-plastic deformation occurs with the progress of each reversal, leading to energy dissipation [23]. This dissipation in energy, depending on its definition, can be related to fatigue damage process. Early energy-based approaches for fatigue life prediction which relate the hysteresis energy or plastic strain energy to fatigue life can only be applied to completely reversed stress condition, as these approaches are basically insensitive to mean stress ($\sigma_{m}$) [24]. To address this limitation, Golos and Ellyin [25] modified the model and defined the total strain energy $\left(∆W^{t}\right)$ as the summation of plastic strain energy $\left(∆W^{p}\right)$ and elastic strain energy $\left(∆W^{e}\right)$:

(1) $$∆W^{t}=∆W^{p}+∆W^{e}$$

For a Masing-type material, $∆W^{p}$ which is the area of a hysteresis loop can be expressed as follows:

(2) $$∆W^{p}=\frac{1-n^{\prime }}{1+n^{\prime }}∆\sigma ∆\varepsilon^{p}$$

In this equation, $n^{\prime }$ is the cyclic strain-hardening exponent. The stress range (∆σ) is related to plastic strain range $\left(∆\varepsilon^{p}\right)$ according to the Ramberg–Osgood equation:

(3) ∆ε2=∆εe2+∆εp2=∆σ2E+(∆σ2H′)1n′

In this equation, $H^{\prime }$ is cyclic strength coefficient and E is the elastic modulus. In general, the fatigue failure criterion can be expressed as follows:

(4) $$∆W^{t}=\frac{1-n^{\prime }}{1+n^{\prime }}∆\sigma ∆\varepsilon^{p}+∆W^{e}=W_{e}^{\prime }(N_{f}{)}^{B}+W_{f}^{\prime }(N_{f}{)}^{C}$$

In the above equation, $W_{e}^{\prime }$ and $W_{f}^{\prime }$ are fatigue strength and fatigue toughness coefficients, and *B * and *C * are fatigue strength and fatigue toughness exponents, respectively. As mentioned earlier, accurate definitions of elastic and plastic strain energy densities are essential for energy-based fatigue life predictions. While the plastic strain energy is widely accepted as the area within a stabilized hysteresis loop, there is ongoing debate regarding the appropriate definition of elastic strain energy. It is worth noting that the model proposed by Golos and Ellyin [25] is limited to loading conditions with negative minimum stress. To address mean stress effects across a broader range of conditions, including both positive and negative minimum stress values, researchers have developed alternative approaches. According to a model proposed by Jahed and Varvani [14] the summation of both terms of positive and negative elastic strain energies has been considered as the total elastic strain energy:

(5) $$∆W^{e}=2\oint H\left(\sigma_{x}\right)H(d\varepsilon_{xx}^{e})\sigma_{x}d\varepsilon_{xx}^{e}$$

In which H(x) is a Heaviside function which is defined as follows:

(6) $$\left\{\begin{array}{c} H\left(x\right)=1\;for\;x\geq 0\\ H\left(x\right)=0\;for\;x<0 \end{array}\right.$$

The proposed model has demonstrated its effectiveness, leading to reasonable results across a wide range of metallic materials subjected to both proportional and non-proportional multi-axial loading conditions. Koh [26] introduced a formulation that correlates the stress range with the elastic strain energy density as follows:

(7) $$∆W^{e}=\frac{1}{2E}(\sigma_{max}-\sigma_{min}{)}^{2}=\frac{{∆\sigma }^{2}}{2E}$$

Dallmeier et al. [27] introduced a method for calculating the elastic strain energy density that considers the variation between maximum and minimum strain energy density terms during a cycle. Since compressive stresses are less effective than tensile stresses in contributing to fatigue degradation under cyclic loading, researchers have suggested considering only the positive portion of the strain energy density (SED) for fatigue life predictions [17,28]. Total strain energy density, comprising both positive elastic and plastic components, has been shown to yield fatigue life predictions closely aligned with experimental data. Lin et al. [28] proposed the following formulation for the positive elastic strain energy density term:

(8) $$∆W^{e+}=\left\{\begin{array}{c} \frac{\sigma_{max}^{2}(1-R{)}^{2}}{2E}\;\sigma_{min}\geq 0\\ \frac{\sigma_{max}^{2}}{2E}\;\sigma_{min}\leq 0 \end{array}\right.$$

Fan defined the positive elastic energy term depending on different values of stress ratio R [28]:

(9) $$∆W^{e+}=\left\{\begin{array}{c} \frac{∆\sigma^{2}}{2E}+\sigma_{min}∆\varepsilon \;\;\;\;\;\;\;\;\;\;\;\;\;\;\;\;\;\;\;\;\;\;\;\;\;\;\;\;\;\;\;\;\;\;\;\;\;\;\;\;\;R\geq 0\\ \frac{\sigma_{max}^{2}}{2E}+\frac{2n^{\prime }\sigma_{max}}{1+n^{\prime }}(\frac{\sigma_{max}}{2H^{\prime }}{)}^{1/n^{\prime }}\;\;\;\;\;\;\;\;\;\;\;\;-1\leq R\leq 0\\ \frac{\sigma_{max}^{2}}{2E}\;\;\;\;\;\;\;\;\;\;\;\;\;\;\;\;\;\;\;\;\;\;\;\;\;\;\;\;\;\;\;\;\;\;\;\;\;\;\;\;\;\;\;\;\;\;\;\;\;\;\;\;\;\;R\leq -1 \end{array}\right.$$

**Figure 1:**
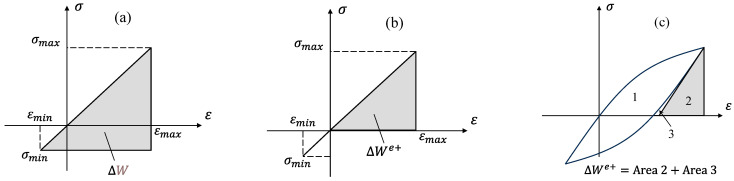
Definitions of elastic and positive elastic SED in energy-based fatigue models: (**a**) Koh [26], (**b**) Lin et al. [28], and (**c**) Fan [28].

Figure 1 schematically illustrates how the aforementioned models define the elastic and positive elastic SED terms.

Roostaei et al. [18] came up with an idea that a single term across all ranges of stress ratio value can be used for fatigue life prediction. Their proposed model is as follows:

(10) $$∆W^{e+}=\frac{(\sigma_{max}+\sigma_{m})\sigma_{a}}{2E_{cyc}}$$

where $\sigma_{max}$ and $\sigma_{min}$ are the maximum and minimum stresses, and *E* is the Young modulus. In this study, the four aforementioned models (Equations (7)–(10)) were evaluated for predicting the fatigue life of 3D-printed samples. It is worth noting that numerical calculations were used to obtain the elastic and plastic strain energy density (SED) values of the filaments and 3D-printed samples for stabilized cycles.

## 3. Experimental Data

**Figure 2:**
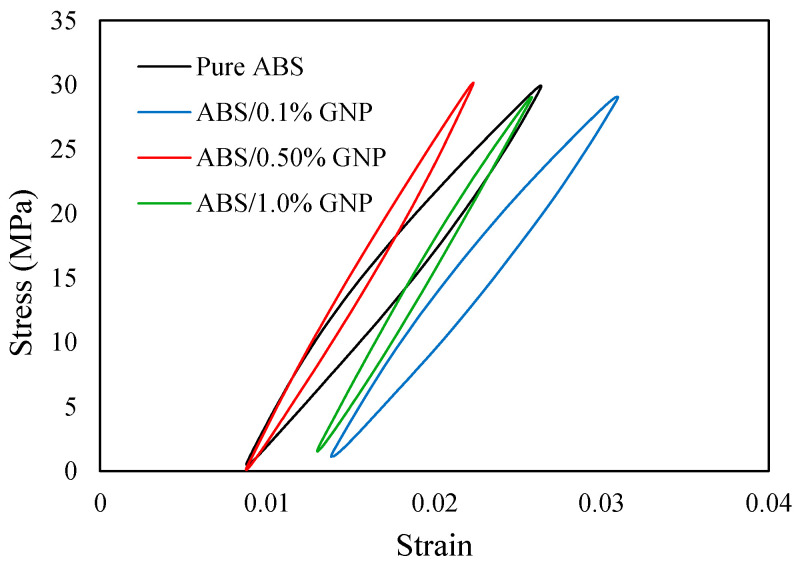
Hysteresis loops at a stabilized cycle for the filaments subjected to 30 MPa load.

The material selected for this study is ABS polymer (purchased from 3DXTECH, Grand Rapids, MI, USA) reinforced with varying concentrations of GNPs (purchased from ACS Material, LLC, Pasadena, CA, USA) at 0.1, 0.5, and 1.0 wt.%. ABS was selected due to its widespread use in FDM 3D printing, offering good processability and mechanical properties. GNPs were incorporated as a filler to enhance the mechanical performance of ABS. The mechanical behavior of the nanocomposite filament and 3D-printed specimens was assessed through quasi-static and fatigue testing, conducted in accordance with ASTM D638-22 [29] and ASTM D7791-22 [30], respectively. For tensile testing, Type IV dog-bone specimens, as defined by ASTM D638 [29], were utilized to ensure consistency with standard procedures. The highest standard deviation in stress values obtained from the quasi-static tests was 2.4 MPa, which corresponds to the filament containing 1.0 wt.% GNP. Details on filament and 3D-printed sample fabrication, as well as quasi-static and fatigue test data, can be found in reference [31]. It is worth mentioning that the cyclic tests of the filaments have been carried out at stress ratio of 0≤R<0.05 at different stress levels ranging approximately between 40 to 80% the materials’ ultimate tensile strength (UTS). During cyclic loading, the filament materials exhibited gradual degradation in mechanical properties, particularly a reduction in Young’s modulus by approximately 4–15%, along with a ratcheting behavior. An average degraded Young modulus value was used in this study to obtain the required stress and strain values for the 3D-printed samples. Figure 2 shows the experimentally obtained hysteresis loops at a stabilized cycle for the filaments under a typical load level of 30 MPa, highlighting differences in their shape and corresponding strain energy density values.

Further details on the cyclic behavior of the filaments are available in Ref. [32]. A summary of the required data and parameters for this study is presented in Table 1.

To apply energy-based fatigue life prediction models, it is essential to first plot the hysteresis loop and obtain the necessary stress and strain values, including stress range (or mean stress) and strain amplitude. These values can be determined by simultaneously solving two sets of Ramberg–Osgood and Neuber’s equations for each load level. Neuber’s equation is given as follows:

(11) $$\sigma \varepsilon =\frac{(K_{f}^{\prime }\;S{)}^{2}}{E}$$

**Figure 3:**
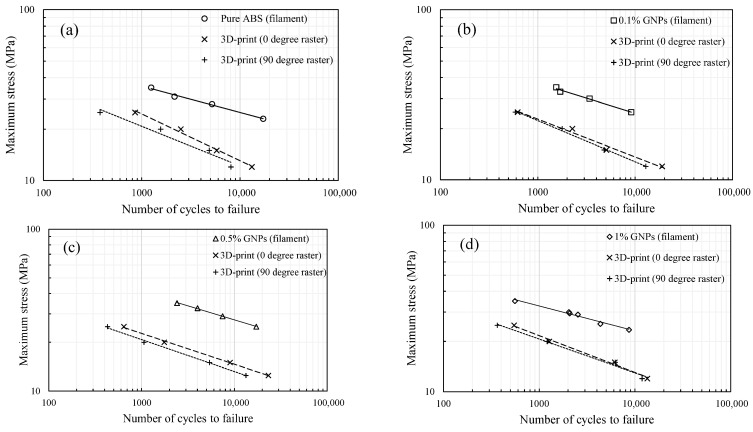
Fatigue test data of filaments and 3D-printed samples, (**a**) Pure ABS, (**b**) ABS/0.1% GNP, (**c**) ABS/0.5%GNP, and (**d**) ABS/1.0%GNP [31].

In this equation, σ and ε are the local stress and strain value at the notch root or at the location of stress concentration for a notched specimen, and $K_{f}^{\prime }$ and *S* denote the notch strength reduction factor and applied remote stress, respectively. Experimental results indicate that the fatigue life of the 3D-printed samples was significantly lower than that of the filaments with the same GNP content [31]. This suggests that porosity and gaps between filaments act as stress concentrators. To account for this, the 3D-printed samples were modeled as homogeneous, isotropic components containing an imaginary notch, with notch strength reduction factors determined at various load levels. The imaginary notch concept idealizes the stress concentration effect of inter-filament voids in FDM parts. It is conceptually assumed to be elliptical, with the major axis oriented perpendicular to the loading direction. While its geometry is not explicitly modeled or assigned fixed dimensions, the notch is defined such that its root radius produces a notch strength reduction factor similar to that of the actual printed part. Figure 3 summarizes the fatigue test data for 3D-printed samples with 0° and 90° raster orientations, along with those of the filaments [31].

From the above plots and fatigue test data, the notch strength reduction factors can be determined using the following formula:

(12) $$K_{f}^{\prime }=\frac{\sigma_{smooth}}{\sigma_{notch}}$$

In this equation, $\sigma_{smooth}$ and $\sigma_{notch}$ represent the stress values applied to the filament as a smooth material and to the 3D-printed samples as notched specimens at a given number of fatigue life cycle. The notch strength reduction factors for specimens with raster angles of 0° and 90° were directly extracted from experimental fatigue test data and applied in fatigue life predictions. The observed trend in these notch factors suggested that values for other raster orientations could be estimated through an interpolation between the 0° and 90° data. Specifically, the relation $K_{f}^{\prime }=1.46(\sigma /UTS{)}^{-0.22}$ was found to provide a good fit for samples with a 45° raster orientation. Consequently, for these samples, the notch strength reduction factors were used without requiring experimental fatigue test data.

## 4. Results and Discussion

### 4.1. Energy-Life Material Properties

**Figure 4:**
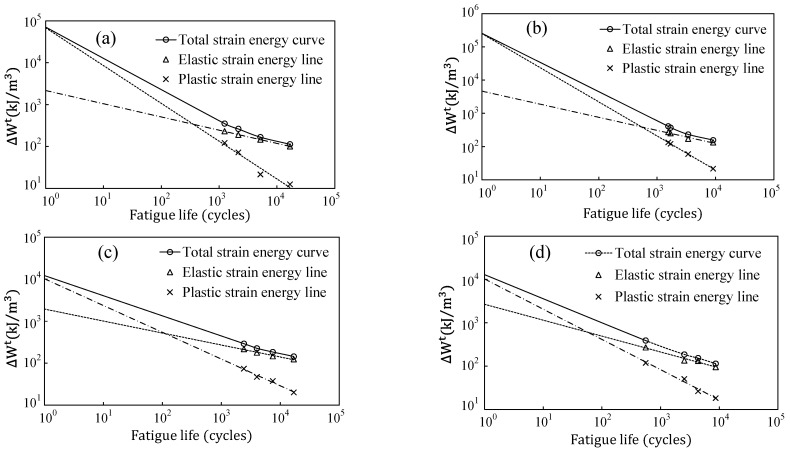
Energy-life parameter extraction using total energy density versus fatigue life curves; (**a**) Pure ABS, (**b**) ABS/0.1% GNP, (**c**) ABS/0.5%GNP, and (**d**) ABS/1.0%GNP.

Fatigue test data can be used to obtain energy-based fatigue parameters of the filament composites. By evaluating the total strain energy density, which is the sum of both plastic and elastic energy densities, these parameters can be obtained through the curve fit of two-term power law of total energy-life of Equation (4). For consistency and given that the stress ratio R≅0, the elastic strain energy term was evaluated using ${∆W}^{e}=\sigma_{max}^{2}/2E$, while the plastic energy term was derived from the hysteresis loop area. Figure 4 illustrates the parameter extraction process using stress-controlled fatigue test data and corresponding total energy density values. The energy-based fatigue parameters of the filament materials are summarized in Table 2.

### 4.2. Application to 0° and 90° Raster Angles

Fatigue life prediction of 3D-printed parts was performed for three raster angles: 0°, 45°, and 90°. Predicting fatigue life requires stress/strain responses and corresponding energy density values, obtained by solving Equations (3) and (11) for each loading condition at maximum stress and stress amplitude. For 0° and 90° raster orientations, experimentally determined notch strength reduction factors were used. The computed energy density values were then applied to fatigue life predictions using the energy-based models in Equations (7)–(10).

**Figure 5:**
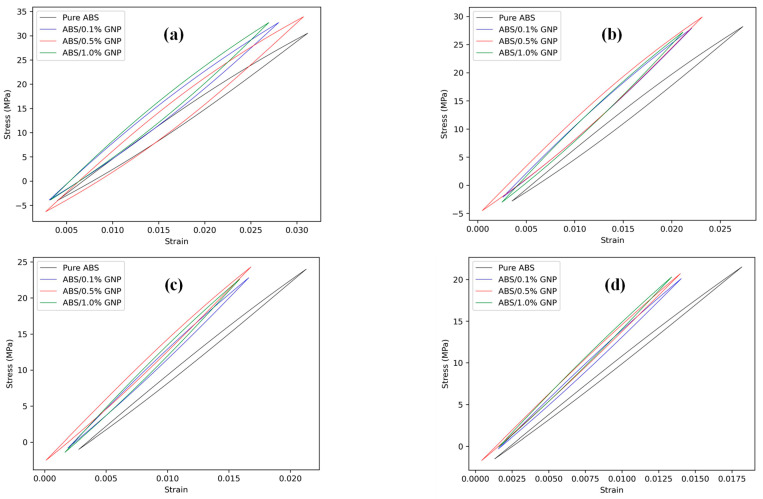
The stress/strain responses of 3D-printed samples undergoing maximum nominal stress of (**a**) 25 MPa, (**b**) 20 MPa, (**c**) 15 MPa, and (**d**) 12 MPa for the samples with 0° raster orientation.

**Figure 6:**
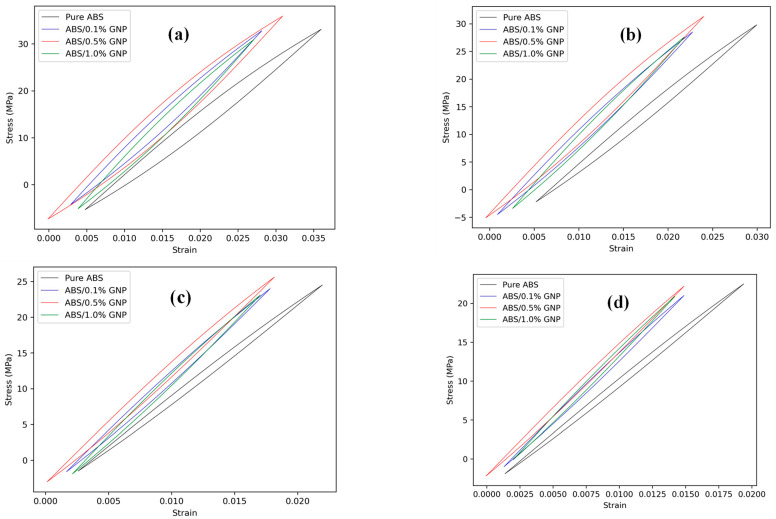
The stress/strain responses of 3D-printed samples undergoing maximum nominal stress of (**a**) 25 MPa, (**b**) 20 MPa, (**c**) 15 MPa, and (**d**) 12 MPa for the samples with 90° raster orientation.

Figure 5 and Figure 6 illustrate the stress/strain responses (hysteresis loops) for all applied loading conditions in 0° and 90° raster orientations, respectively. These figures show that variations in GNP content significantly affect the stress/strain response, influencing energy density terms and, consequently, fatigue life. Additionally, 3D-printed samples with a 90° raster orientation exhibited higher elastic and plastic strain energy densities due to their relatively higher notch strength reduction factors, as observed in Figure 3.

In particular, the ABS/0.1% GNP filaments exhibited a slight deviation from the general trend observed in other compositions. This behavior may be attributed to interconnected factors such as variations in yield strength, differences in plastic hardening behavior, and the presence of potential agglomeration, voids, or internal defects introduced during processing.

**Figure 7:**
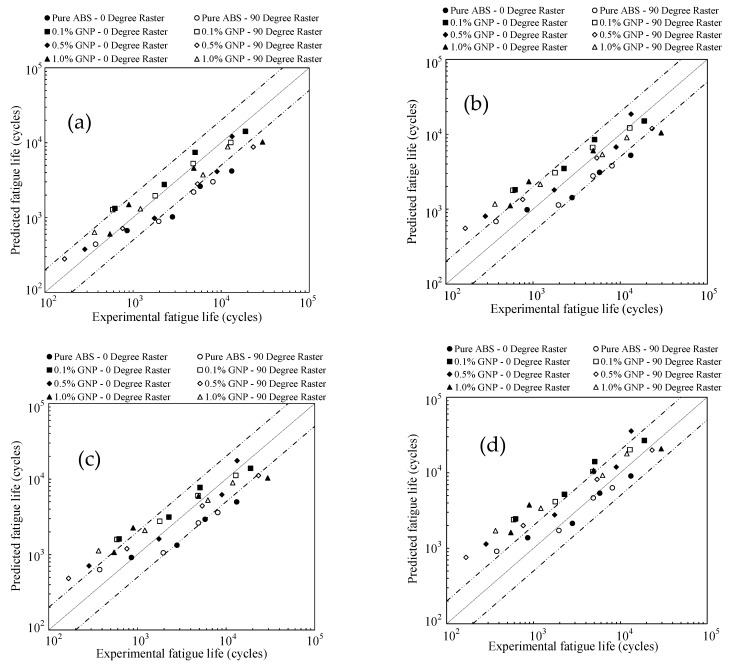
Fatigue life predictions of 3D-printed samples using the studied energy-based models for 0° and 90° raster orientations; (**a**) Koh [26], (**b**) Lin et al. [28], (**c**) Fan [28], and (**d**) Roostaei et al. [18].

The data required for fatigue life predictions, extracted from these figures, is summarized in Table 3. Figure 7 presents the fatigue life predictions of 3D-printed samples using the studied energy-based models. The results indicate that the models proposed by Lin et al. [28] and Fan [28] overpredict fatigue life at higher load levels and slightly underpredict it at lower load levels. In contrast, Koh [26] model provides better predictions at higher load levels. The model by Roostaei et al. [18] tends to overpredict fatigue life, particularly at high load levels, but offers relatively better accuracy at lower load levels compared to other models.

### 4.3. Application to 45° Raster Angle

**Figure 8:**
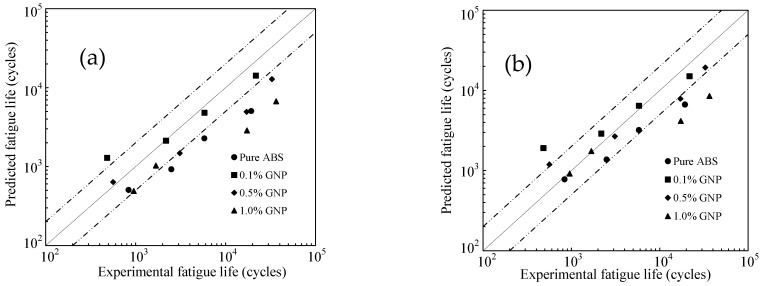

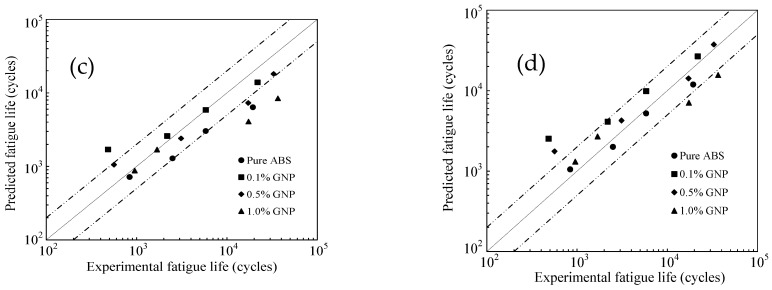
Fatigue life predictions of 3D-printed samples using the studied energy-based models for 45° raster orientation; (**a**) Koh [26], (**b**) Lin et al. [28], (**c**) Fan [28], and (**d**) Roostaei et al. [18].

The same procedure was applied to predict the fatigue life of 3D-printed samples with a 45° raster orientation. However, instead of actual experimentally obtained values, the estimated notch strength reduction factors were used. The necessary stress/strain and energy density data are summarized in Table 4, while Figure 8 presents the fatigue life predictions for these samples. The overall trend follows a similar pattern to that observed in Figure 7. While some models exhibit deviations at different load levels, the general tendencies remain consistent with those seen for 0° and 90° orientations. This approach ensures consistency in the analysis while accounting for the influence of raster orientation and the application of different energy-based models on the fatigue behavior of additively manufactured composite parts.

### 4.4. Proposed Approach

The results for all raster orientations from the previous sections indicate that none of the studied energy-based models accurately predicted fatigue life across the entire range of stress ratio values. As the load level decreases (with R increasing from a minimum of −0.22 to approximately 0), the models proposed by Lin et al. [28] and Fan [28] shift from overpredicting fatigue life to underpredicting it. In contrast, Roostaei et al. [18] model provides reliable predictions for R≥−0.1, while Koh [26] model is more accurate within the range of −0.22≤R<−0.1. Given these observations, it is reasonable to divide the negative mean stress values into two regions to improve prediction accuracy. The proposed model incorporates both Koh [26] and Roostaei et al. [18] approaches by segmenting the studied R values into the following two regions:

(13) $$∆W^{e+}=\left\{\begin{array}{c} \frac{(\sigma_{max}+\sigma_{m})\sigma_{a}}{2E_{cyc}}\;\;\;\;\;\;\;\;\;\;\;\;\;\;\;\;R\geq -0.1\\ \frac{1}{2E}(\sigma_{max}-\sigma_{min}{)}^{2}\;\;\;\;R<-0.1 \end{array}\right.$$

**Figure 9:**
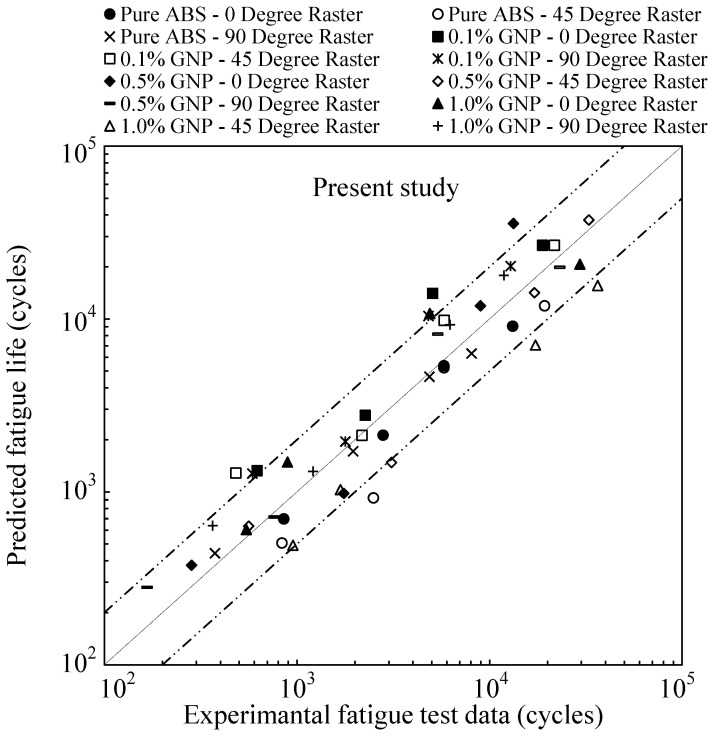
Fatigue life predictions of 3D-printed samples using the proposed approach for all raster orientations.

Figure 9 presents the fatigue life predictions for samples with all studied raster orientations using the proposed energy-based approach. The results demonstrate the method’s effectiveness, as most of the data points fall within the twice-error band.

The findings of the present study underscore the limitations of existing energy-based models in the literature, suggesting that further refinement is necessary to improve accuracy based on specific material properties and loading conditions. Dallmier et al. [27] observed that the positive elastic energy term, similar to that in Lin et al.’s model [28], overestimated the influence of mean stress on total fatigue damage. To address this, they introduced a weighting coefficient of 0.25 to refine the total strain energy density equation as follows [27]:

(14) $$∆W^{t}=∆W^{p}+0.25∆W^{e+}$$

However, this correction may lead to an overestimation of fatigue life, particularly under loading conditions with high negative stress ratio values. The challenge arises because while adjusting the total strain energy density equation improves predictions for certain cases, it does not fully account for the complex interactions between material behavior, mean stress effects, and fatigue damage accumulation. Zhu et al. [33] similarly noted the limitations of using total strain energy density to properly account for mean stress effects in fatigue life prediction and proposed a method to overcome this issue. As a result, the accuracy of fatigue life prediction remains an open area of research, requiring further investigation and refinement of existing models to enhance their reliability across a broader range of loading conditions and materials.

## 5. Conclusions

This study explored the capability of energy-based models in predicting the fatigue life of additively manufactured ABS/GNP composites, considering the influence of different raster orientations and GNP weight fractions. By employing Neuber and Ramberg–Osgood equations, stress and strain values were determined using notch strength reduction factors and effective Young’s modulus for each composite. The analysis demonstrated that existing energy-based models struggled to provide accurate fatigue life predictions across both high- and low-cycle fatigue regimes, particularly due to their sensitivity to the stress ratio (R). To address this limitation, a new fatigue prediction approach was formulated by integrating two established models, effectively accounting for negative mean stress conditions. Validation of this approach showed that the majority of predicted fatigue lives for all raster orientations fell within a ±2 error factor, confirming its improved reliability compared to existing methods. These findings emphasize the necessity of refining fatigue models to better capture the complex stress interactions in 3D-printed composite materials under varying loading conditions.

## Figures and Tables

**Table 1:** Mechanical properties and strain-hardening parameters of the studied filaments [31].

GNP Content (%)	E (MPa)	E* (MPa)	Yield Strength (MPa)	UTS (MPa)	$H^{\prime }$	$n^{\prime }$
0 (Pure ABS)	1643	1438	17.8	37.0	127.3	0.31
0.1	1942	1760	18.2	43.8	192.4	0.38
0.5	2081	1751	18.1	39.8	128.7	0.31
1.0	2095	1867	21.1	37.8	99.4	0.24

E* is the average value of the degraded Young modulus in a stabilized cycle.

**Table 2:** Energy-life parameters of the filament materials.

Material	$W_{e}^{\prime }(MJ/m^{3})$	$W_{f}^{\prime }(MJ/m^{3})$	B	C
Pure ABS	2.17	67.98	−0.32	−0.90
ABS/0.1% GNP	4.65	248.68	−0.39	−1.02
ABS/0.5% GNP	1.94	10.24	−0.29	−0.64
ABS/1.0% GNP	2.66	10.23	−0.36	−0.69

**Table 3:** Stress/strain and energy-life parameters of the 3D-printed composites with 0° and 90° raster angles.

Material	Nominal Stress (MPa)	Raster Angle (°)	Stress/Strain Data					${∆W}^{e+}(kJ/m^{3})$				${∆W}^{p}$ $(kJ/m^{3})$
			$\sigma_{max}$	$\sigma_{a}$	R	$\varepsilon_{max}$	$\varepsilon_{a}$	Lin et al. [28]	Koh [26]	Roostaei et al. [18]	Fan [28]	
Pure ABS	25	0	30.5	17.2	−0.13	0.0311	0.0156	323.4	411.4	262.8	338.8	53.7
		90	33.1	19.2	−0.16	0.0357	0.0134	380.9	512.7	313.7	402.6	79.1
	20	0	28.2	15.5	−0.09	0.0270	0.0117	276.5	334.1	220.4	287.5	34.9
		90	29.6	16.0	−0.08	0.0304	0.0123	308.7	356.0	242.5	322.7	40.7
	15	0	24.0	12.5	−0.04	0.0213	0.0096	200.2	217.3	154.3	205.8	14.6
		90	24.5	13.0	−0.06	0.0220	0.0108	208.7	235.0	162.7	214.8	17.1
	12	0	21.5	11.5	−0.07	0.0182	0.0085	160.7	183.9	125.9	164.2	10.1
		90	22.5	12.2	−0.08	0.0198	0.0095	176.0	207	139.1	180.0	13.7
ABS/0.1% GNP	25	0	32.7	18.3	−0.12	0.0280	0.0125	303.7	380.5	244.8	331.2	63.1
		90	32.8	18.5	−0.13	0.0282	0.0128	305.6	388.9	247.5	333.3	65.1
	20	0	27.9	15.5	−0.11	0.0222	0.0107	221.1	255.6	173.8	236.5	31.9
		90	28.5	16.5	−0.16	0.0228	0.0110	230.7	309.3	189.8	247.4	41.6
	15	0	22.8	11.8	−0.04	0.0168	0.0083	147.6	158.2	113.3	155.1	13.5
		90	24.0	12.8	−0.07	0.0178	0.0084	163.6	186.2	128.0	172.5	17.9
	12	0	20.1	10.2	−0.01	0.0139	0.0070	114.7	118.2	86.9	119.4	8.0
		90	21.0	11.0	−0.05	0.0149	0.0071	125.2	137.5	96.8	130.7	10.5
ABS/0.5% GNP	25	0	33.9	20.1	−0.19	0.0329	0.0139	328.1	484.7	273.7	351.4	92.0
		90	35.9	21.6	−0.22	0.0367	0.0154	368.0	532.9	309.6	397.6	121.8
	20	0	29.9	17.2	−0.15	0.0261	0.0113	255.2	337.9	209.2	268.9	49.7
		90	31.3	18.2	−0.16	0.0283	0.0122	279.7	378.3	230.7	296.3	62.2
	15	0	24.4	13.4	−0.10	0.0185	0.0083	168.6	205.1	134.6	174.3	18.1
		90	25.8	14.2	−0.10	0.0201	0.0089	187.1	233.5	150.7	194.2	23.6
	12	0	20.7	11.2	−0.08	0.0145	0.0067	122.3	143.2	96.6	125.2	8.6
		90	22.2	12.1	−0.09	0.0161	0.0073	140.7	167.2	116.0	144.6	11.9
ABS/1.0% GNP	25	0	31.2	18.2	−0.17	0.0247	0.0107	260.6	354.8	215.4	266.0	33.3
		90	30.9	18.0	−0.17	0.0242	0.0104	255.7	347.1	211.1	260.8	31.5
	20	0	27.2	15.1	−0.11	0.0191	0.0088	198.1	244.2	158.9	200.7	13.5
		90	27.6	15.5	−0.12	0.0196	0.0090	204.0	257.3	164.7	206.8	15.2
	15	0	22.6	12.0	−0.06	0.0142	0.0066	136.7	154.2	106.7	137.8	4.3
		90	23.1	12.5	−0.08	0.0146	0.0073	142.9	167.3	112.8	144.0	5.2
	12	0	20.3	10.2	0.00	0.0122	0.0056	110.3	111.4	83.0	110.9	1.8
		90	20.9	10.5	0.00	0.0127	0.0063	116.9	118.1	88.0	117.6	2.2

**Table 4:** Stress/strain and energy-life parameters of the 3D-printed composites with 45° raster orientation.

Material	Nominal Stress (MPa)	Stress/Strain Data					${∆W}^{e+}(kJ/m^{3})$				${∆W}^{p}$ $(kJ/m^{3})$
		$\sigma_{max}$	$\sigma_{a}$	R	$\varepsilon_{max}$	$\varepsilon_{a}$	Lin et al. [28]	Koh [26]	Roostaei et al. [18]	Fan [28]	
Pure ABS	25	32.1	18.5	−0.15	0.0342	0.0148	358.3	476.0	293.9	377.3	69.8
	20	28.3	15.9	−0.12	0.0275	0.0122	278.4	351.6	225.0	289.6	38.0
	15	23.7	12.9	−0.09	0.0209	0.0096	195.3	231.4	154.7	200.6	16.2
	12	20.5	10.9	−0.06	0.0171	0.0080	146.1	165.2	114.1	148.9	8.1
ABS/0.1% GNP	25	33.4	18.8	−0.13	0.0289	0.0128	292.7	388.9	240.2	318.4	63.4
	20	29.1	16.1	−0.11	0.0233	0.0106	240.6	294.5	192.6	258.5	40.1
	15	24.1	13.1	−0.09	0.0179	0.0083	165.0	195.0	130.6	174.1	19.3
	12	20.1	10.2	−0.01	0.0139	0.0070	114.7	118.2	86.9	119.4	8.0
ABS/0.5% GNP	25	31.9	18.6	−0.17	0.0294	0.0126	290.6	395.1	240.0	308.5	67.9
	20	28.1	16.0	−0.14	0.0235	0.0103	225.4	292.4	183.6	235.9	37.1
	15	23.7	13.0	−0.10	0.0177	0.0081	160.4	193.0	127.7	165.5	16.1
	12	20.6	11.1	−0.08	0.0144	0.0067	121.1	140.7	95.4	124.0	8.3
ABS/1.0% GNP	25	32.3	19.0	−0.18	0.0265	0.0113	279.4	386.7	232.0	285.8	41.0
	20	28.7	16.3	−0.14	0.0211	0.0093	220.6	284.6	179.4	224.1	19.5
	15	24.3	13.2	−0.09	0.0158	0.0073	158.1	186.6	125.1	159.6	6.9
	12	21.1	11.1	−0.05	0.0129	0.0061	119.2	131.9	92.4	119.9	2.9

## Data Availability

The original contributions presented in this study are included in the article. Further inquiries can be directed to the corresponding author.
